# Sex‐differences in network level brain dynamics associated with pain sensitivity and pain interference

**DOI:** 10.1002/hbm.25245

**Published:** 2020-10-17

**Authors:** Junseok A. Kim, Rachael L. Bosma, Kasey S. Hemington, Anton Rogachov, Natalie R. Osborne, Joshua C. Cheng, Benjamin T. Dunkley, Karen D. Davis

**Affiliations:** ^1^ Division of Brain, Imaging and Behaviour Krembil Brain Institute, Krembil Research, Institute, University Health Network Toronto Ontario Canada; ^2^ Institute of Medical Science, Faculty of Medicine University of Toronto Toronto Ontario Canada; ^3^ Department of Medical Imaging University of Toronto Toronto Ontario Canada; ^4^ Neurosciences & Mental Health Program The Hospital for Sick Children Research Institute Toronto Ontario Canada; ^5^ Department of Surgery University of Toronto Toronto Ontario Canada

**Keywords:** functional connectivity, magnetoencephalography, network dynamics, pain, sex differences

## Abstract

Neural dynamics can shape human experience, including pain. Pain has been linked to dynamic functional connectivity within and across brain regions of the dynamic pain connectome (consisting of the ascending nociceptive pathway (Asc), descending antinociceptive pathway (Desc), salience network (SN), and the default mode network (DMN)), and also shows sex differences. These linkages are based on fMRI‐derived slow hemodynamics. Here, we utilized the fine temporal resolution of magnetoencephalography (MEG) to measure resting state functional coupling (FCp) related to individual pain perception and pain interference in 50 healthy individuals (26 women, 24 men). We found that pain sensitivity and pain interference were linked to within‐ and cross‐network broadband FCp across the Asc and SN. We also identified sex differences in these relationships: (a) women exhibited greater within‐network static FCp, whereas men had greater dynamic FCp within the dynamic pain connectome; (b) relationship between pain sensitivity and pain interference with FCp in women was commonly found in theta, whereas in men, these relationships were predominantly in the beta and low gamma bands. These findings indicate that dynamic interactions of brain networks underlying pain involve fast brain communication in men but slower communication in women.

## Graphical abstract

We used magnetoencephalography to investigate network‐level brain dynamics that may underlie individual variability in pain behavior and also, sex differences in this system. We found that pain sensitivity and pain interference were related to within‐ and cross‐network static and dynamic functional coupling across nodes of the ascending nociceptive pathway and the salience network and that there were sex differences in these relationships. These results highlight the brain dynamics underlying pain processing that differ between sexes and neural frequency bands.

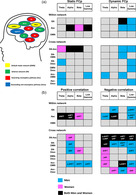

## 1 INTRODUCTION

The brain is a dynamic system that can respond to external stimuli and sustains cognitive and sensory functions that fluctuate throughout everyday situations. It is now understood that variability and fluctuations of brain activity may constitute “priors” that contribute to the perceptual variability within and across people (Kucyi & Davis, 2015, 2017; Mayhew, Hylands‐White, Porcaro, Derbyshire, & Bagshaw, 2013; Ohara, Crone, Weiss, Kim, & Lenz, 2008; Ploner, Gross, Timmermann, Pollok, & Schnitzler, 2006; Ploner, Lee, Wiech, Bingel, & Tracey, 2010). The idea of priors builds on the concept that the brain is intrinsically dynamic (Fox & Raichle, 2007) and this dynamic brain activity observed prior to administration of a stimulus has been linked to different aspects and variability in our perception of somatosensory, auditory, and visual stimuli, cognitive performance, and pain (Boly et al., 2007; Coste, Sadaghiani, Friston, & Kleinschmidt, 2011; Dunkley, Freeman, Muthukumaraswamy, & Singh, 2013; Hesselmann, Kell, & Kleinschmidt, 2008; Sadaghiani, Hesselmann, & Kleinschmidt, 2009).

How such neural dynamics contribute to pain at the individual level is not understood. Pain is a subjective experience and accordingly, there is significant inter‐individual variability in sensitivity, perception, and impact of pain on cognitive functions. The brain mechanisms underlying these features are thought to involve a set of brain regions that dynamically engage with one another, known as the dynamic pain connectome that consists of the ascending nociceptive pathway (Asc), salience network (SN), default mode network (DMN), and descending antinociceptive pathway (Desc) (Kucyi & Davis, 2015, 2017). Therefore, here we have examined neural dynamics in the dynamic pain connectome to gain insight into the individual variability of the human pain experience.

The dynamics of neural activity within and between brain networks can be examined using an fMRI approach known as dynamic functional connectivity (Hutchison et al., 2013). Our previous studies used this technique to establish the concept of the dynamic pain connectome and subsequently found links between dynamic functional connectivity, pain sensitivity, mind wandering, and the effect of pain on cognitive task performance (i.e., the “A”/“P” phenotype) during painful stimulation (Cheng et al., 2017; Erpelding & Davis, 2013; Kucyi, Salomons, & Davis, 2013). These studies established, the importance of dynamics in brain communication pertaining to pain but the slow hemodynamics (seconds timescale) of fMRI restricted such studies. In the current study, we have examined neural dynamics on a millisecond timescale that is in keeping with true temporal fidelity of neuronal activity using magnetoencephalography (MEG). The MEG technique allowed us to investigate functional coupling at different frequencies. Such phase and amplitude measures can provide mechanistic insight about communication within the brain (Buzsaki, 2006; Siegel, Donner, & Engel, 2012). Thus in the current study, we were interested in using MEG to examine the relationship between aspects of pain (pain sensitivity, and pain interference on cognitive performance) and oscillatory neural communication in the brain.

It is becoming increasingly evident that sex differences contribute to the individual variability in pain perception and its underlying neural mechanism. For example, previous studies have demonstrated sex differences in pain thresholds and suprathreshold responses (Berkley, 1997; Greenspan et al., 2007; Hashmi & Davis, 2014; Jensen et al., 2011) and in functional connectivity within regions of the dynamic pain connectome, especially in the salience network and in the descending antinociceptive pathway (Coulombe, Erpelding, Kucyi, & Davis, 2016; Rogachov et al., 2016; Wang, Erpelding, & Davis, 2014). Furthermore, there are sex differences in the prevalence of many chronic pain conditions (Pardue & Wizemann, 2001; Unruh, 1996) and in the response to some treatments (Bartley & Fillingim, 2013; Fillingim, King, Ribeiro‐Dasilva, Rahim‐Williams, & Riley III, 2009). However, how brain dynamics, specifically in network‐level oscillatory communication within the dynamic pain connectome, contribute to these sex differences is not understood.

Therefore the aims of this study are to determine: (a) Is neural oscillatory communication within the dynamic pain connectome related to pain sensitivity and pain interference on cognitive performance and (b) Are there sex differences in oscillatory communication within the dynamic pain connectome, and if so, are these related to pain sensitivity or pain interference on cognitive performance.

## 2 MATERIALS AND METHODS

### 2.1 Participants

**FIGURE 1:**
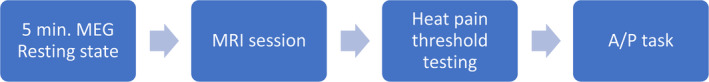
Overview of the neuroimaging and psychophysical testing protocols

We recruited 50 people between the ages of 18–39 (24 men, age (mean ± *SD*) 27 ± 5, 26 women, age (mean ± *SD*) 28 ± 6) through posted advertisements and word of mouth. Informed written consent was collected from all participants of the study for the study protocol approved by the research ethics board of the University Heath Network. The inclusion criteria were: (a) no prior history of chronic pain or current experience of pain on a regular basis, (b) free of metabolic, psychiatric or neurologic conditions, (c) no history of major surgery due to a physiological condition, (d) no contraindications for the MRI, and (e) age under 40 years old. An overview of the study design is shown in Figure 1.

### 2.2 Heat pain threshold determination

Each participant's heat pain threshold (HPT) was determined using the method of limits. Heat stimuli were delivered to the participant's volar forearm approximately 15 cm from the wrist using a 30 × 30 mm thermode (TSA, Medoc Inc.). For each stimulus trial, the baseline thermode temperature was 32°C from which the temperature increased at a ramp rate of 0.5°C/s until the participant pressed a mouse button or if the thermode reached the maximum point at 50°C, at which point the thermode temperature returned to baseline at a rate of 2°C/s. Each participant was instructed to click a mouse button at the first moment that they felt the heat stimulus was painful. For each participant, three trials of HPT determination was performed with an inter trial time of 5 s and the average temperature of the last two trials was deemed to be the HPT.

### 2.3 Effect of pain on a cognitive test

To define the effect of pain on a cognitive task, we performed a task which was previously described and used (Cheng et al., 2017; Erpelding & Davis, 2013; Kucyi et al., 2013; Seminowicz & Davis, 2006, 2007; Seminowicz, Mikulis, & Davis, 2004). The cognitive task is a modified version of the numerical task (Eccleston & Crombez, 1999; Windes, 1968) that has been utilized instead of the Stroop task because it has been shown to produce robust differences in reaction time (RT) between task conditions (Erpelding & Davis, 2013). Briefly, participants viewed a computer screen that displayed three boxes, each containing multiple copies of a digit from 1 to 9. Each of the three boxes contained a different number of copies of digits and unique value of digits in each box. For example, box one might have three 2 s, box two might have seven 5 s, and box three might have nine 1 s. The participant is instructed to use a numerical keypad to report the number of digits in the box that contains the most number of digits. The instructions are to respond as quickly and as accurately as possible. The outcome measure of the task was the RT for each correct trial. The value of the number and the number of digits inside each box was different across each trial. Each participant underwent 6 blocks of the task which alternated between pain and no pain blocks (3 pain blocks and 3 no pain blocks) with the no pain block always being the first block. Every block contained 24 trials with each trial lasting for 2.5 s. Between each block, there was a short interval of 20 s and a 5 s countdown was provided to indicate the start of the next block.

Pain was evoked by transcutaneous electrical nerve stimulation (TENS) of the median nerve at the left wrist area. The intensity of the painful stimulation was determined prior to the task to induce a pain rated as 40–50 (0 = no pain, 100 = worst pain imaginable). Although some habituation could have occurred for some individuals, the intensity of the stimulus was high enough that pain intensity remained in this range throughout the testing. After the task, a pain intensity rating of 40–50 was confirmed in each participant.

For our outcome measures of interest, we first used the method outlined in a previous paper (Cheng et al., 2017) to model each individuals' RT to the task as an ex‐Gaussian function, a convolution of a Gaussian and an exponential function (Ratcliff, 1979). This was performed to account for outliers in the mean and standard deviation calculations of the RT resulting from lapses in attention (Heathcote, Popiel, & Mewhort, 1991) which can inflate mean and standard deviation values of RT (Ratcliff, 1993). From this the mean, the standard deviation and the exponential component was extracted. We confined our analysis to the last two blocks (no‐pain and pain) consisting of 24 trials in each condition because we previous observed learning effects present in the first four blocks (Cheng et al., 2017). We also removed incorrect trials (i.e., not reporting the correct number of digits in the box with the most amount of digits or failing to report a value within 2.5 s) and omitted participants who had low accuracy rates (i.e., <75% correct trials). We quantified the interference effect based on the difference of the mean RT values between the pain and no‐pain blocks. Individuals who have disrupted cognitive performance due to pain were classified as P‐type individuals (i.e., pain dominant) and individuals who performed better with painful stimulation were classified as A‐type individuals (i.e., attention dominant) as per our previous studies (Cheng et al., 2017; Erpelding & Davis, 2013; Seminowicz et al., 2004). The difference of mean RT was always calculated with mean RT no pain block—mean RT pain block. Individuals with negative mean RT difference had improved cognitive performance during painful stimulation (i.e., A‐type individual) and individuals who had positive mean RT difference had worse cognitive performance during painful stimulation (i.e., P‐type individual).

### 2.4 Neuroimaging data acquisition

Every participant underwent a 5 min “resting state” scan using the Elekta Neuromag TRIUX system using a 1,000 Hz sampling rate and bandpass filter of 330 Hz at recording. To minimize artifacts, participants removed any metallic objects they wore and were asked to refrain from wearing make‐up or hair products which may have metallic residue. For co‐registration to individual's structural MRI and real‐time movement detection purposes, cardinal points were marked at the nasion, right, and left preauricular positions and five head position coils were placed around the head. The location of cardinal points and head position coils were identified using an electronic positioning system. Once placed inside the darkened magnetically‐shielded room, each participant was asked to view a cross‐hair displayed on a screen in front of the scanner, and to let their mind wander freely for the duration of the resting state scan. Artifact correction was performed using the MaxFilter program. Participants also underwent a 3T MRI (GE Medical Systems, Chicago, IL) high resolution T1 scan of the brain (1 × 1 × 1 mm^3^ voxels, matrix = 256 × 256, FOV = 25.6, flip angle 15°, 180 axial slices, repetition time = 7.8 s, echo time = 3 ms, inversion time = 450 ms) so that the MEG data could be co‐registered brain anatomy.

### 2.5 MEG data preprocessing and beamforming

The preprocessing pipeline that was used for the MEG data was based on the MATAB program FieldTrip (http://www.fieldtriptoolbox.org/). We applied a bandpass filter between 1 and 150 Hz and a notch filter at 60 and 120 Hz. Independent components analysis using “runica” was used to remove artefactual components likely cause by respiration, heartbeats and eye‐blinks. The MEG resting state data was co‐registered to each individual's high resolution T1 image using the cardinal points obtained during MEG data collection. The forward model was constructed using a single‐shell model (Nolte, 2003).

Time‐series data from “virtual sensors” were extracted from regions of interest (ROIs) using the atlas‐guided beamforming method (Hillebrand, Barnes, Bosboom, Berendse, & Stam, 2012). The Linearly Constrained Minimum Variance beamformer (Van Veen, van Drongelen, Yuchtman, & Suzuki, 1997) was used for this purpose and a time‐series was reconstructed for each ROI at the center of mass. The beamformer is used to isolate signals of interest while suppressing signals from unwanted sources. For each source estimated in the brain, a weighting vector is applied to the signals obtained from the physical sensors. The reconstructed time‐series from the previously defined ROIs is a result of the summation of all the time‐series that are estimated for each ROI.

### 2.6 Regions of interest

The ROIs within key components of the dynamic pain connectome were selected for the atlas‐guided beamforming. The ROIs were chosen based on our previous MEG study (Kim et al., 2019): (a) Asc: left primary somatosensory cortex (S1) (−34, −30, 54), right S1 (34, −28, 54), left secondary somatosensory cortex (S2) (−60, −30, 20), right S2 (60, −22, 18), left and right posterior insula (−/+34, −20, 18), left and right thalamus (−/+12, −18, 8); (b) SN: right temporoparietal junction (TPJ) (50, −32, 28), right anterior insula (34, 18, 4), mid cingulate cortex (MCC) (2, 12, 34), right dorsolateral prefrontal cortex (dlPFC) (34, 46, 22); (c) DMN: posterior cingulate cortex (PCC) (−2, −46, 28), and medial prefrontal cortex (mPFC) (−2, 50, 2); (d) Desc: subgenual anterior cingulate cortex (sgACC) (4, 26, –8).

### 2.7 Indices of functional coupling

We used two metrics to assess functional coupling (FCp): amplitude envelope correlation (AEC) (Z. Liu, Fukunaga, de Zwart, & Duyn, 2010) and weighted phase lag index (wPLI) (Vinck, Oostenveld, van Wingerden, Battaglia, & Pennartz, 2011). We used the mathematical formula in MATLAB to calculate these metrics. AEC is calculated by correlating the band‐filtered amplitude envelope between time‐series of each pair of ROIs and this measure has been most closely associated with fMRI measures of functional connectivity (Brookes et al., 2011; De Pasquale et al., 2010; Z. Liu et al., 2010). wPLI is a metric used to quantify the amount of nonzero phase lag between two ROIs, defining the consistency of phase synchrony between the two regions. In cross‐regional oscillatory interactions, phase and amplitude are believed to provide different information regarding the underlying neurophysiology and can in part act independently of one another (Siegel et al., 2012). Instantaneous phase and amplitude was calculated for each time point in the time‐series extracted from each ROI using the Hilbert Transform. A finite impulse response filter was used to filter data in the canonical frequency bands: theta (4–8 Hz), alpha (8–13 Hz), beta (13–30 Hz), low gamma (30–60 Hz), and high gamma (60–150 Hz). To correct for the presence of source leakage we used orthogonalization in the ROI‐nets toolbox (Colclough, Brookes, Smith, & Woolrich, 2015). The process of orthogonalization removes linear dependencies from two time‐series by taking a set of nonorthogonal independent functions and creating an orthogonal relationship between them. Orthogonalization is important to attenuate “ghost” coupling between two ROIs in amplitude envelope correlation (Palva et al., 2018). The first and last 10 s were removed from each resting state scan resulting in 280 s of data. This was then divided into 28 10 s epochs.

We calculated both static and dynamic FCp (sFCp, dFCp) values for each participant using the following approach: Static FCp was calculated as the mean of all the epoched FCp values, whereas epoched FCp values were calculated within 10 second epochs. Dynamic FCp can measure the fluctuation of FCp between two regions over time. To calculate dynamic FCp, a standard deviation was calculated from the FCp values across the 28 10 s epochs.

### 2.8 Statistical testing

Group differences in amplitude envelope correlation, wPLI, HPT, and pain interference were based on two‐tailed Student's *t*‐tests between the male and female groups. The Benjamin‐Hochberg method (Benjamini & Hochberg, 1995) at *p* < .05 was used to correct for multiple comparisons for the number of nodes. Correlation between FCp and HPT as well as pain interference were performed using Spearman's correlation with significance set at *p* < .05, corrected for multiple comparisons with the Benjamin‐Hochberg method.

## 3 RESULTS

### 3.1 Behavioral data

The HPT findings were based on 49 out of the 50 participants (24 men 25 women) because there was a thermode malfunction during testing of one subject. The pain interference findings were based on data from 46 out of 50 participants (22 men 24 women) because there was a TENS malfunction during testing of four participants.

The behavioral data are summarized in Table 1. The average HPT was 41.5 ± 3.8°C (range, 35.1–47.8°C) and the mean pain interference was −69.26 ± 109.6 ms (range, −329.3 to 130.7 ms, 33 A‐type individuals and 14 P‐type individuals). There were no sex differences in age (*p* = .33, mean ± *SD* men 27 ± 5 years old and women 28 ± 6 years old) or pain interference (*p* = .86, mean ± *SD* men −67.8 ± 103.1 ms, women −70.56 ± 117.5 ms). However, HPT was significantly lower in women (mean ± *SD* 40.2 ± 3.6°C) compared to men (mean ± S.D. 42.8 ± 3.7°C; *p* = .014).

**TABLE 1:** Behavioral data of the participants

	Whole group	Male	Female
*N*	50	24	26
Age	27 ± 5 [18–39]	27 ± 5 [18–39]	28 ± 6 [19–36]
HPT (°C)	41.5 ± 3.8 [35.1–47.8]	42.8 ± 3.7^a^ [35.1‐47.8]	40.2 ± 3.6 [35.6–47.5]
Pain interference (ms)	−69.26 ± 109.6 [−329.3–130.7]	−67.8 ± 103.1 [−236.5–121.4]	−70.56 ± 117.5 [−329.3–130.7]
Number of A/P type	33 A; 14 P	16 A; 7 P	17 A; 7 P

*Note:* Data shown are mean ± *SD* and range. Values inside the square bracket indicate minimum and maximum value.

Abbreviation: HPT, heat pain threshold.

^a^ Group differences at *p* < .05.

### 3.2 HPT is correlated with broadband cross‐network functional coupling

In this study we have used two different measures of FCp (AEC and wPLI) that describe phase‐ or amplitude‐based communication in the brain. Furthermore, static and dynamic variants of the FCp measures are able to capture the average strength of this connection (static) compared to the amount of fluctuation in the communication (dynamic). As such, the combination of the FCp metric and their variants can reveal the dynamic interaction within the dynamic pain connectome.

**FIGURE 2:**
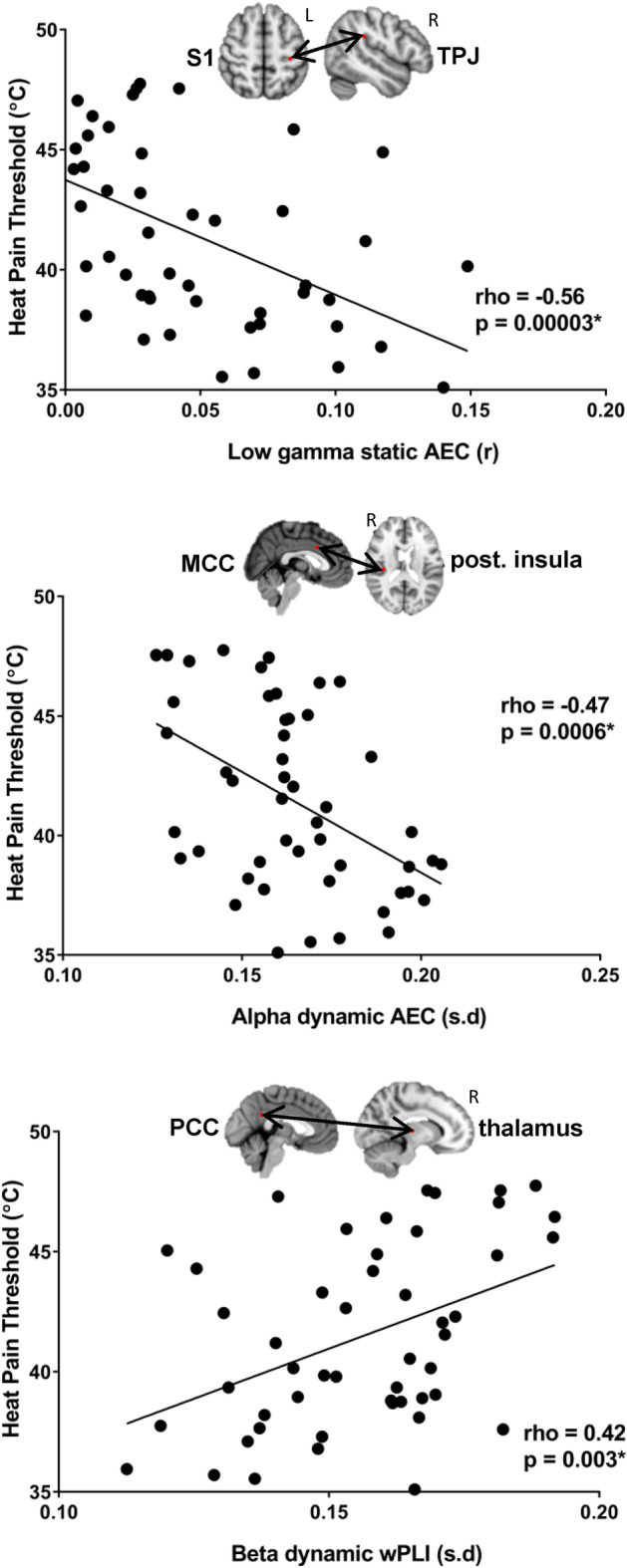
Examples of significant correlation between static and dynamic FCp and heat pain threshold. The example nodes from left to right: cross‐network static gamma amplitude envelope correlation between the left primary somatosensory cortex and the right temporoparietal junction, cross‐network dynamic alpha amplitude envelope correlation between the mid‐cingulate cortex and the right posterior insula, dynamic beta weighted phase lag index between the posterior cingulate cortex and the right thalamus. AEC, amplitude envelope correlation; MCC, mid‐cingulate cortex; PCC, posterior cingulate cortex; S1, primary somatosensory cortex; TPJ, temporoparietal junction; wPLI, weighted phase lag index

The whole group analysis of AEC in the SN, Asc, and DMN revealed that HPT is correlated with cross‐network static and dynamic FCp in the theta, alpha, beta and low gamma bands (for a summary of findings, see Table 2). Figure 2 provides some representative examples of these correlations.

**TABLE 2:** Summary of correlations between heat pain threshold, pain interference, and functional coupling

	Heat pain threshold					Pain interference			
	Theta	Alpha	Beta	Low gamma		Theta	Alpha	Beta	Low gamma
**Within network**									
SN			sFCp	sFCp		sFCp			
Asc	dFCp			sFCp		sFCp	s,dFCp		sFCp
DMN									
**Cross network**									
SN‐Asc	dFCp	s,dFCp	s,dFCp	s,dFCp		s,dFCp	sFCp	dFCp	s,dFCp
SN‐DMN	dFCp	s,dFCp				sFCp			
SN‐Desc									dFCp
DMN‐Desc									
Asc‐DMN		s,dFCp	dFCp	sFCp					
Asc‐Desc			dFCp					dFCp	

*Note:* sFCp and dFCp mark significant correlations present with static and dynamic functional coupling. Significance determined at FDR < .05.

Abbreviations: Asc, ascending nociceptive pathway; d, dynamic; Desc, descending antinociceptive pathway; DMN, default mode network; FCp, functional coupling; s, static; SN, salience network.

Within‐network Asc static and dynamic inter‐hemispheric AEC was negatively correlated with HPT in theta and low gamma bands between right S1‐left post. Insula (rho = −0.37, *p* = .008) as well as between the left S1 and right thalamus (rho = −0.4, *p* = .004), right S2 (rho = −0.39, *p* = .005), and right posterior insula (rho = −0.39, *p* = .005). Cross‐network static and dynamic AEC was negatively correlated with HPT in alpha, beta, and low gamma bands Asc‐SN between right posterior insula‐MCC (rho = −0.47, *p* = .0006; Figure 2) as well as between left S1 and the right anterior insula (rho = −0.37, *p* = .009 (beta), rho = −0.35, *p* = .015 (low gamma)), right TPJ (rho = −0.56, *p* = .0003; Figure 2) and the MCC (rho = −0.34, *p* = .015). Cross‐network alpha dynamic and static AEC is negatively associated with HPT between Asc‐DMN between right thalamus‐PCC (rho = −0.37, *p* = .007), and the bilateral posterior insula and mPFC (rho = −0.42, *p* = .002 (left), rho = −0.39, *p* = .006 (right)) and also between SN‐DMN across right TPJ‐mPFC (rho = −0.37, *p* = .009). Cross‐network beta dynamic AEC between Asc‐Desc was negatively associated with HPT across left S2‐sgACC (rho = −0.42, *p* = .003) and left posterior insula‐sgACC (rho = −0.42, *p* = .003).

Also in the whole group, cross‐network theta, alpha, beta, and gamma static and dynamic wPLI was correlated with HPT. Cross‐network static and dynamic wPLI in theta and alpha bands were positively correlated with HPT across SN‐Asc between the right anterior insula‐right S1 (rho = 0.37, *p* = .009) and the right thalamus (rho = 0.38, *p* = .006) however HPT was negatively correlated with beta and low gamma static and dynamic wPLI across SN‐Asc between the right anterior and posterior insula (rho = −0.4, *p* = .004), right TPJ‐right S1 (rho = −0.4, *p* = .004), right TPJ‐left thalamus (rho = −0.37, *p* = .009), and MCC‐left S1 (rho −0.43, *p* = .002). Similar patterns were observed with static and dynamic wPLI in the beta and low gamma band across DMN‐Asc where beta dynamic wPLI was positively correlated with HPT between PCC‐right thalamus (rho = 0.43, *p* = .003; Figure 2) and low gamma static wPLI was negatively correlated with HPT between PCC‐left S2 (rho = −0.41, *p* = .003). HPT was also negatively correlated with cross‐network static and dynamic wPLI in the theta and alpha band across DMN‐SN between the mPFC and MCC (rho = −0.41, *p* = .004) as well as right dlPFC (rho = −0.41, *p* = .004). Last, within network static beta wPLI was negatively correlated with HPT across right TPJ‐right anterior insula (rho = −0.43, *p* = .002).

### 3.3 Pain interference is correlated with theta, alpha, and low gamma functional coupling

**FIGURE 3:**
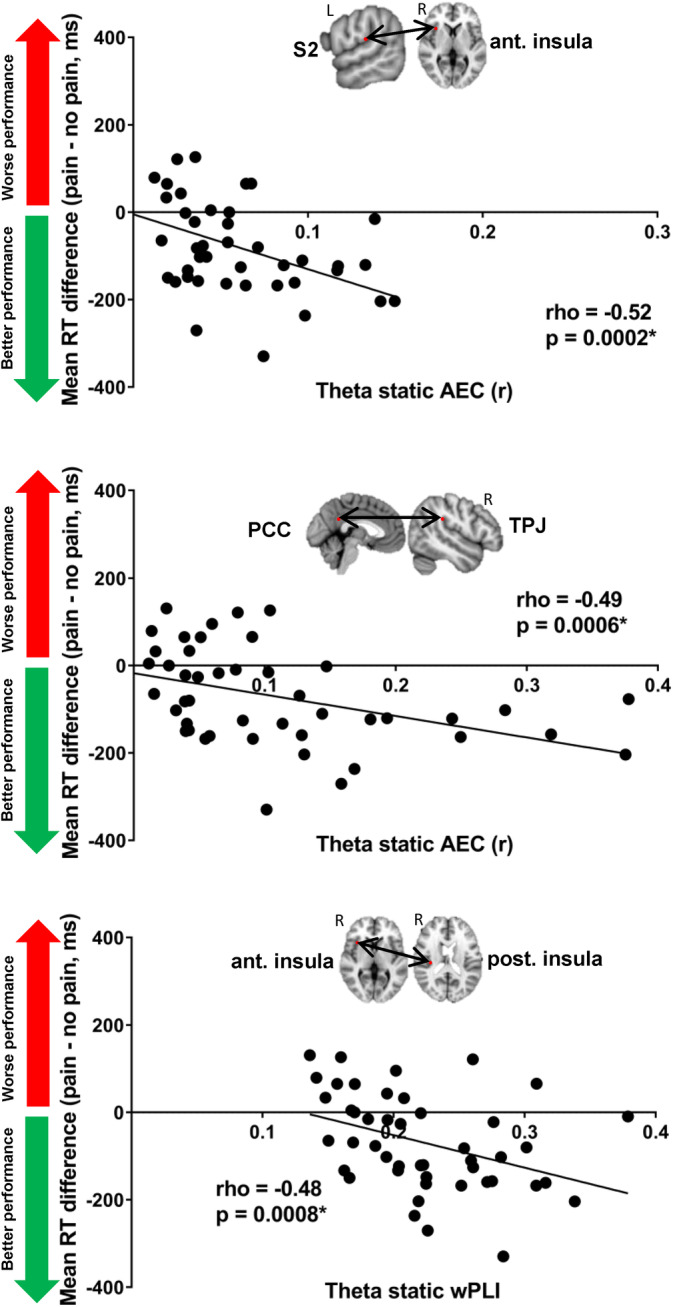
Examples of significant correlation between static and dynamic FCp and pain interference. The example nodes which are shown from left to right: cross‐network static theta amplitude envelope correlation between the left secondary somatosensory cortex and the right anterior insula, cross‐network static theta amplitude envelope correlation between the posterior cingulate cortex and the right temporoparietal junction, static theta weighted phase lag index between the right anterior insula and the right posterior insula. AEC, amplitude envelope correlation; PCC, posterior cingulate cortex; S2, secondary somatosensory cortex; TPJ, temporoparietal junction; wPLI, weighted phase lag index

In the entire cohort, static and dynamic AEC in the theta, alpha, and low gamma bands in both within‐ and cross‐network nodes were correlated with pain interference. All of the correlations are summarized on Table 2 and examples of the correlations are displayed on Figure 3.

Pain interference was negatively correlated with within‐network static AEC in the theta, alpha, and low gamma bands across right thalamus‐left S2 (rho = −0.42, *p* = .004), left S1‐right S1 (rho = −0.4, *p* = .006), left S2‐right S1 (rho = −0.46, *p* = .001), left S2‐right posterior insula (rho = −0.45, *p* = .002), left S1‐right S2 (rho = −0.46, *p* = .001), left thalamus‐left S1 (rho = −0.39, *p* = .009), and right S1‐right posterior insula (rho = −0.38, *p* = .009), however, it was positively correlated with dynamic alpha AEC between left thalamus and left S2 (rho = 0.42, *p* = .004). Cross‐network static and dynamic AEC in theta, alpha, beta, and low gamma was negatively correlated with pain interference across Asc‐SN between right thalamus‐MCC (rho = −0.39, *p* = .007), right thalamus‐right dlPFC (rho = −0.39, *p* = .008 (theta), rho = −0.41, *p* = .005 (low gamma)), left S1‐right anterior insula (rho = −0.4, *p* = .006), left S2‐right anterior insula (rho = −0.52, *p* = .0002) (Figure 3), right S2‐right dlPFC (rho = −0.42, *p* = .004), right posterior insula‐right dlPFC (rho = −0.39, *p* = .007), right S1‐right TPJ (rho = −0.38, *p* = .008), and right anterior and posterior insula (rho = −0.39, *p* = .008). Last, pain interference was negatively correlated with static and dynamic AEC in theta and low gamma bands across DMN‐Asc between mPFC‐right thalamus (rho = −0.41, *p* = .005) and right S2 (rho = −0.39, *p* = .008) as well as SN‐DMN between right TPJ‐PCC (rho = −0.49, *p* = .0006; Figure 3) and between SN‐Desc across right TPJ‐sgACC (rho = −0.37, *p* = .009).

Within‐ and cross‐network static and dynamic wPLI in theta, alpha beta and low gamma bands were also correlated with pain interference. Within network static alpha and low gamma wPLI across nodes of the Asc was negatively correlated with pain interference between left S2‐left thalamus (rho = −0.44, *p* = .002), left S2‐right S2 (rho = −0.39, *p* = .008), left S2‐right posterior insula (rho = −0.46, *p* = .001), right S1‐right S2 (rho = −0.39, *p* = .008), right S2‐left posterior insula (rho = −0.43, *p* = .003), and the left and right posterior insula (rho = −0.38, *p* = .009). As well, pain interference was negatively correlated with within SN dynamic theta wPLI across right TPJ‐right anterior insula (rho = −0.41, *p* = .005) but positively correlated with within DMN static theta wPLI across PCC‐mPFC (rho = 0.38, *p* = .009). Cross‐network static and dynamic theta, low gamma wPLI between the Asc‐SN was negatively correlated with pain interference across right S2‐right anterior insula (rho = −0.39, *p* = .008 (dynamic), rho = −0.46, *p* = .001 (static)), right posterior and anterior insula (rho = −0.48, *p* = .0008) (Figure 3), right thalamus‐right TPJ (rho = −0.39, *p* = .007), left posterior and right anterior insula (rho = −0.43, *p* = .003), left S2‐right TPJ (rho = −0.4, *p* = .005), left S2‐right anterior insula (rho = −0.36, *p* = .01), and also negatively correlated with cross‐network dynamic beta wPLI between Asc‐Desc across the right S1‐sgACC (rho = −0.45, *p* = .002).

### 3.4 Sex differences in functional coupling are most pronounced across cross‐network nodes in the beta and low gamma bands

**FIGURE 4:**
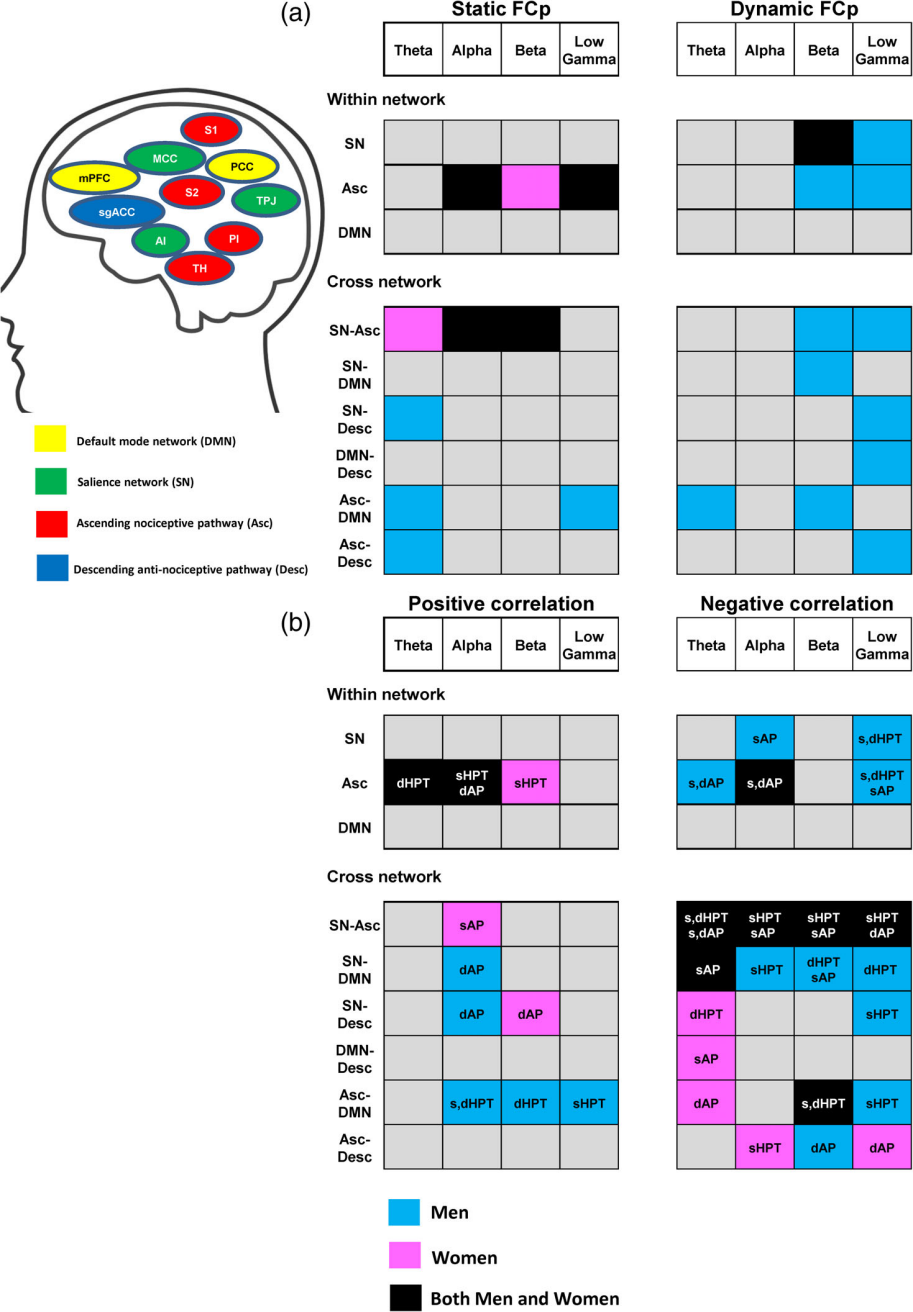
Summary figure of the sex differences analyses for FCp and correlation with pain measures. (a) Significant differences in static and dynamic FCp between men and women with light blue representing greater FCp in men, pink indicating greater FCp in women and black indicating greater FCp in both groups depending on the nodes of the network (b)Different patterns of significant correlations between FCp and heat pain threshold/pain interference in men and women. HPT or AP (pain interference) indicates significant correlations with heat pain threshold and pain interference while s and d indicates static or dynamic FCp, respectively. Light blue indicates significant correlation observed in only men, pink indicates significant correlations in only women and black indicates significant correlations observed in both groups. Significance determined at FDR for *t*‐tests and correlations <.05. Asc, ascending nociceptive pathway; d, dynamic; Desc, descending antinociceptive pathway; DMN, default mode network; FCp, functional coupling; HPT, heat pain threshold; s, static; SN, salience network

**FIGURE 5:**
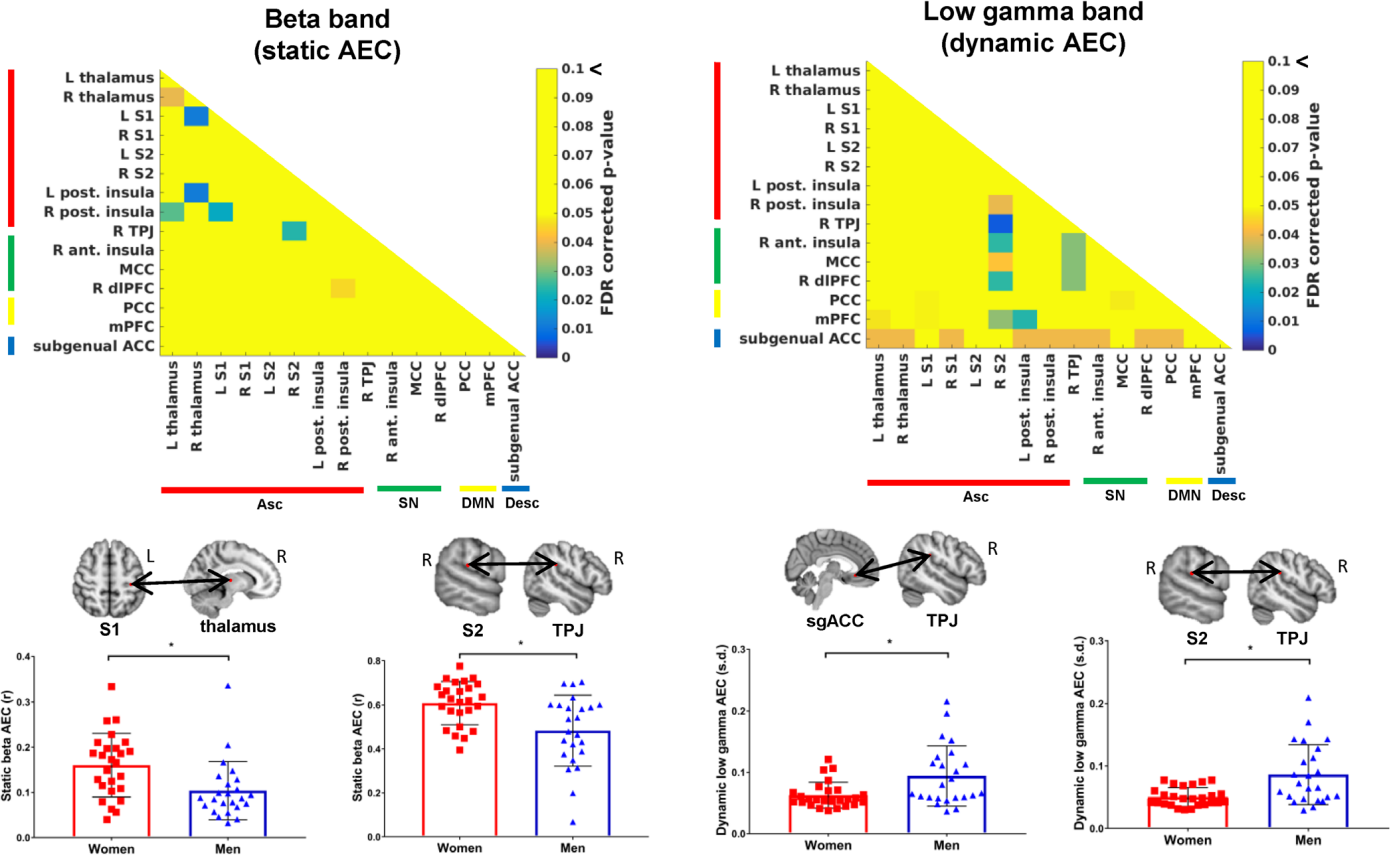
Examples of sex differences in static and dynamic amplitude coupling between men and women. Static beta amplitude envelope coupling differences are shown on the left with the left primary somatosensory cortex‐right thalamus and right secondary somatosensory cortex‐right temporoparietal junction nodes shown as examples. Dynamic low gamma amplitude envelope correlations are shown on the right with the subgenual anterior cingulate cortex‐right temporoparietal junction and right secondary somatosensory cortex‐right temporoparietal junction shown as examples. Significance determined at FDR < .05. AEC, amplitude envelope correlation; Asc, ascending nociceptive pathway; Desc, descending antinociceptive pathway; DMN, default mode network; S1, primary somatosensory cortex; S2, secondary somatosensory cortex; SN, salience network; sgACC, subgenual anterior cingulate cortex; TPJ, temporoparietal junction

**FIGURE 6:**
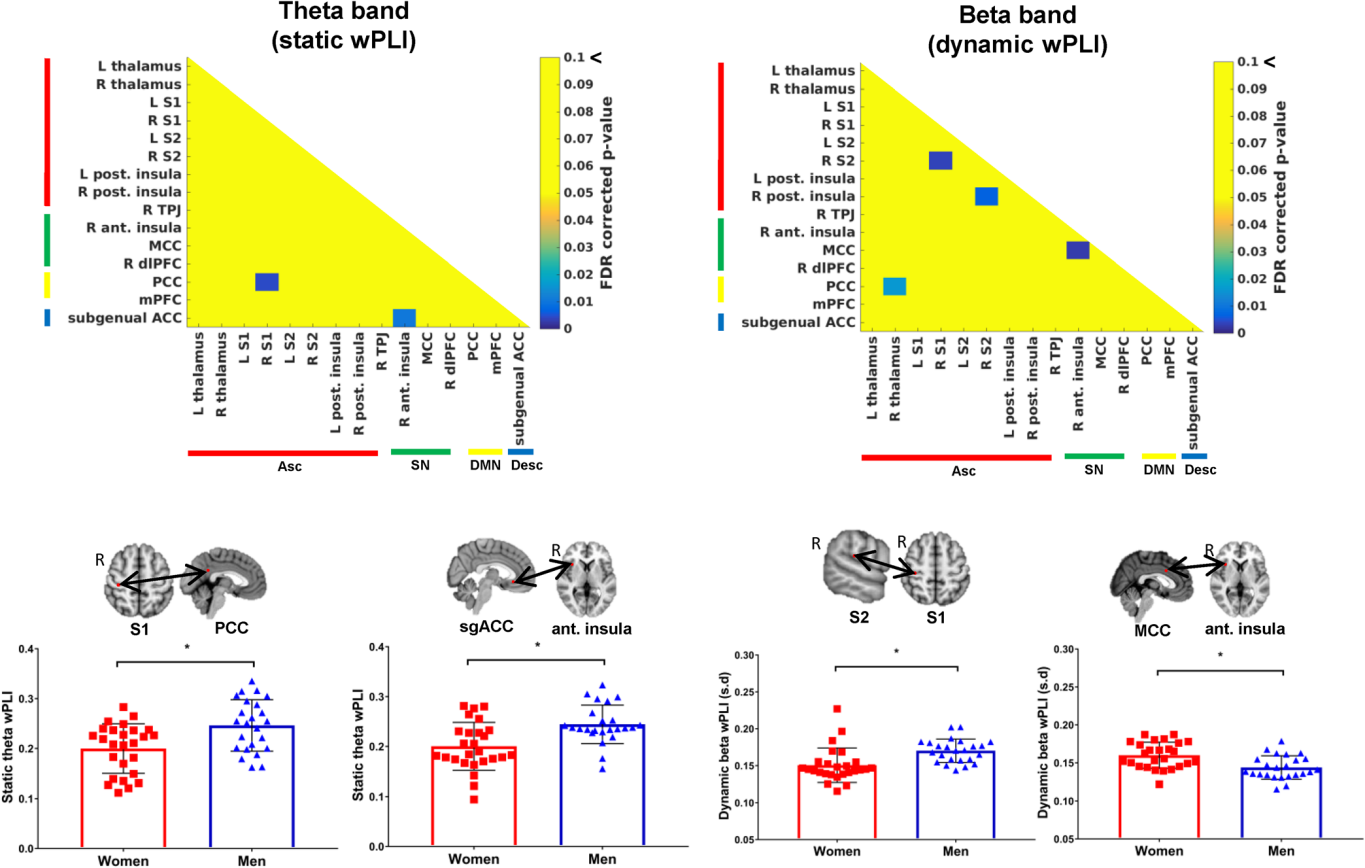
Examples of sex differences in static and dynamic weighted phase lag index between men and women. Static theta weighted phase lag index differences are shown on the left with the right primary somatosensory cortex‐posterior cingulate cortex and subgenual anterior cingulate cortex‐right anterior insula nodes shown as examples. Dynamic beta weighted phase lag index differences are shown on the right with the right secondary somatosensory cortex‐right primary somatosensory cortex and mid‐cingulate cortex‐right anterior insula shown as examples. Significance determined at FDR < .05. Asc, ascending nociceptive pathway; Desc, descending antinociceptive pathway; DMN, default mode network; MCC, mid‐cingulate cortex; PCC, posterior cingulate cortex; S1, primary somatosensory cortex; S2, secondary somatosensory cortex; SN, salience network; sgACC, subgenual anterior cingulate cortex; wPLI, weighted phase lag index

In the sex differences analysis, group differences in static and dynamic AEC were observed across many cross‐network nodes of the dynamic pain connectome. There was a general pattern of greater dynamic AEC in male brains and lower static AEC in female brains. All of the results are summarized on Figure 4a and examples of the correlations are displayed on Figures 5 and 6.

Sex differences were most pronounced across Asc‐SN in theta, alpha, beta, and low gamma bands and these were observed as greater dynamic AEC and less static AEC in male brains between the right S2 node and: the right TPJ (*p* = .001, *p* = .004 (theta dAEC, sAEC), *p* = .0007, *p* = .006 (beta dAEC, sAEC), *p* = .0015 (low gamma); Figure 5), MCC (*p* = .04), right dlPFC (*p* = .017) and the right anterior insula (*p* = .018), as well as left S1‐right TPJ (*p* = .007), right thalamus‐right dlPFC (*p* = .008), and right posterior insula‐right dlPFC (*p* = .01). Other cross‐network nodes that showed greater dynamic low gamma AEC in male brains included: Desc‐Asc between the sgACC node and: left thalamus (*p* = .015), right thalamus (*p* = .028), right S1 (*p* = .009), left posterior insula (*p* = .015), and the right posterior insula (*p* = .026) as well as Desc‐SN between the sgACC node and: right TPJ (*p* = .0066; Figure 5), right anterior insula (*p* = .026) and the right dPFC (*p* = .011), between Desc‐DMN across PCC‐sgACC (*p* = .025) and also between the DMN‐Asc, right TPJ‐PCC (*p* = .0076). Sex differences in within‐network static and dynamic alpha, beta, and low gamma AEC were observed in nodes of the Asc: greater static AEC but less dynamic AEC was observed in female brains compared to male brains between the left S1 node and: the right thalamus (*p* = .009 (alpha sAEC), *p* = .0047 (beta sAEC); Figure 5) and the right posterior insula (*p* = .0036 (alpha sAEC), *p* = .009 (beta sAEC)), left thalamus‐right posterior insula (*p* = .007), right thalamus‐left posterior insula (*p* = .006), right S2‐right posterior insula (*p* = .01), and left thalamus‐right thalamus (*p* = .019). Male brains showed greater dynamic beta and low gamma AEC compared to female brains within nodes of the SN between the right TPJ and the right anterior insula (*p* = .03), MCC (*p* = .02), and the right dlPFC (*p* = .013) as well as between the right anterior insula and the right dlPFC (*p* = .006).

Sex differences in wPLI were most pronounced in the theta, beta, and low gamma bands within the Asc and across Asc‐DMN. Within the Asc, male brains had greater static beta and dynamic gamma wPLI across right S1‐right S2 (*p* = .001) (Figure 6), right S1‐right posterior insula (*p* = .007), and right S2‐right posterior insula (*p* = .0046). Male brains had greater static and dynamic theta, beta, and low gamma wPLI between DMN‐Asc across the PCC node and: right S1 (*p* = .002 (theta dynamic), (*p* = .002 [theta static]; Figure 6), right S2 (*p* = .0055) and the right thalamus (*p* = .008). Male brains also had greater static theta wPLI between nodes of Asc‐Desc between the right anterior insula‐sgACC (*p* = .0007; Figure 6), however, female brains had greater within network dynamic beta wPLI in the SN between the right anterior insula and the MCC (*p* = .001; Figure 6).

### 3.5 Different patterns of relationship observed between HPT and FCp in men and women

**FIGURE 7:**
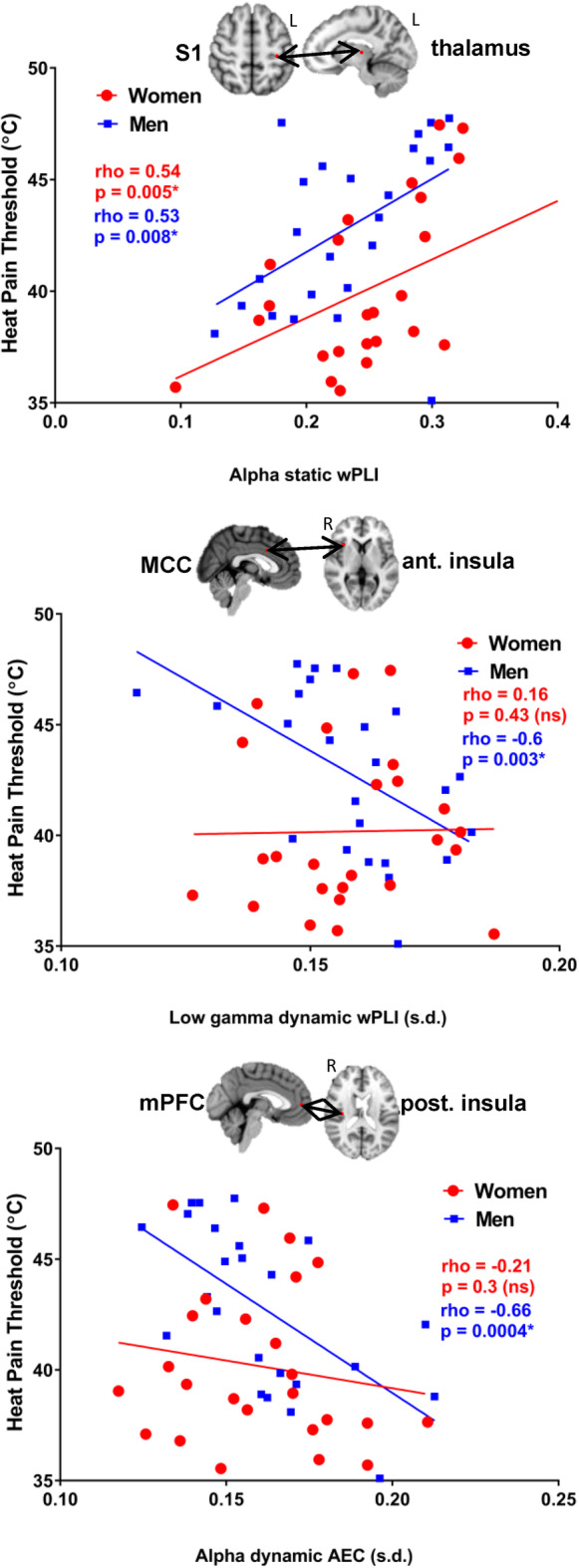
Examples of sex differences in significant correlation between static and dynamic FCp and heat pain threshold. The example nodes which are shown from left to right: within‐network static alpha weighted phase lag index between the left primary somatosensory cortex and the left thalamus, within‐network dynamic low gamma weighted phase lag index between the mid‐cingulate cortex and the right anterior insula, dynamic beta amplitude envelope correlation between the subgenual anterior cingulate cortex and the left posterior insula. AEC, amplitude envelope correlation; MCC, mid‐cingulate cortex; mPFC, medial prefrontal cortex; S1, primary somatosensory cortex; sgACC, subgenual cingulate cortex; wPLI, weighted phase lag index

There were many prominent correlations between HPT and FCp in men where static and dynamic alpha, beta and low gamma FCp was negatively correlated with HPT across several cross‐network nodes of the dynamic pain connectome. Cross‐network static and dynamic alpha, beta and low gamma FCp between SN‐Asc was negatively correlated with HPT across the right dlPFC and: right S1, right S2 and left thalamus as well as right TPJ‐right S2 and right TPJ‐left S2, however theta static and dynamic AEC between SN‐Asc was positively correlated with HPT in right thalamus‐right dlPFC and left S1‐MCC. Cross‐network static and dynamic alpha, beta, and low gamma AEC across DMN‐Asc was negatively correlated with HPT between the mPFC and: the left S1, left posterior insula (Figure 7), right posterior insula and the right S2. HPT was also negatively correlated with cross‐network static low gamma AEC between Desc‐Asc nodes sgACC and: the right S2 and the left posterior insula as well as between nodes of Desc‐SN sgACC‐right TPJ. Last, HPT was negatively correlated with low gamma static FCp across within‐network nodes of Asc and SN: right thalamus‐left S1, right TPJ‐right dlPFC, and right anterior insula‐MCC (Figure 7).

In women, there were both negative and positive correlations between static and dynamic theta, alpha, beta, and low gamma FCp and HPT. Cross‐network FCp between SN‐Asc in theta, beta, and low gamma static and dynamic FCp were negatively correlated with HPT across the right TPJ and: the right S1, the left S1, and the left posterior insula. Cross‐network dynamic theta and beta FCp between Desc‐Asc as well as Desc‐SN was negatively correlated with HPT across the sgACC and: the left posterior insula and the right dlPFC. Interestingly, within‐network dynamic and static theta, alpha, and beta FCp in the Asc were positively correlated with HPT across nodes of left S2‐right posterior insula, left S1‐right S1 and left S1‐left posterior insula.

In both groups, static alpha wPLI within the Asc nodes of left thalamus and left S1 was positively correlated with HPT (Figure 7).

All of the correlation values are summarized on Table 3 and on Figure 4b and examples of correlations are summarized in Figure 7.

**TABLE 3:** Sex differences in correlations between HPT and FCp

Within‐network	Network nodes	Male HPT (rho)	Female HPT (rho)
Asc	R S1‐L S1	−.54 (LG dwPLI)	.56 (beta swPLI)
	R S1‐R thalamus	−.54 (alpha swPLI)	Ns
	L S1‐L thalamus	.53 (alpha swPLI)	.54 (alpha swPLI)
	L S1‐R thalamus	−.53 (LG sAEC)	Ns
	L S1‐L PI	Ns	.51 (alpha swPLI)
	L S2‐R PI	Ns	.53 (theta dAEC)
	R PI‐R thalamus	.53 (theta dwPLI)	Ns
	L PI‐L thalamus	.54 (beta dwPLI)	Ns
SN	R TPJ‐ R AI	−.56 (LG sAEC)	Ns
	R TPJ‐R dlPFC	−.57 (LG swPLI)	Ns
	R AI‐MCC	−.6 (LG dwPLI)	Ns

Cross‐network			
Asc‐SN	R S1‐R dlPFC	−.56 (LG sAEC) −.63 (LG swPLI) −.64 (LG dwPLI)	Ns
	R S1‐R TPJ	Ns	−.62 (beta swPLI)
	L S1‐R TPJ	Ns	−.58 (LG sAEC)
	L S1‐R dlPFC	−.69 (alpha sAEC)	Ns
	L S1‐MCC	.56 (theta dwPLI)	Ns
	R S2‐R dlPFC	−.56 (LG swPLI) −.67 (LG dwPLI)	Ns
	R S2‐R TPJ	−.56 (LG sAEC)	Ns
	L S2‐ R TPJ	−.61 (beta dwPLI)	Ns
	L PI‐R TPJ	Ns	−.58 (theta dAEC)
	R thalamus‐R dlPFC	.57 (theta swPLI)	Ns
	L thalamus‐R dlPFC	−.53 (alpha dwPLI)	Ns
Asc‐DMN	L S1‐mPFC	−.55 (beta dAEC) −.56 (LG sAEC)	Ns
	R S2‐mPFC	−.6 (LG sAEC) −.59 (beta swPLI)	Ns
	R PI‐mPFC	−.66 (alpha dAEC)	Ns
	L PI‐mPFC	−.59 (alpha sAEC)	Ns
Asc‐Desc	R S2‐sgACC	−.58 (LG sAEC)	Ns
	L PI‐sgACC	−.54 (LG sAEC)	−.6 (beta dAEC)
	R thalamus‐sgACC	−.63 (swPLI)	Ns
SN‐DMN	R dlPFC‐PCC	−.59 (alpha dwPLI)	Ns
SN‐Desc	R TPJ‐sgACC	−.53 (LG sAEC)	Ns

*Note:* Significant Pearson's correlations at FDR < .05 are shown for each group.

Abbreviations: AEC, amplitude envelope correlation; AI, anterior insula; Asc, ascending nociceptive pathway; d, dynamic; Desc, descending antinociceptive pathway; dlPFC, dorsolateral prefrontal cortex; DMN, default mode network; L, left; LG, low gamma; MCC, medial cingulate cortex; PI, posterior insula; R, right; s, static; S1, primary sensory cortex; S2, secondary sensory cortex; sgACC, subgenual anterior cingulate cortex; SN, salience network; TPJ, temporoparietal junction.

### 3.6 Sex differences in the relationship between pain interference and FCp observed in cross‐network nodes in women and within‐network nodes in men

In men, pain interference was negatively correlated with within‐ and cross‐network static and dynamic FCp mainly involving the Asc and the SN. Within‐network theta, alpha, and low gamma static and dynamic FCp were negatively correlated with pain interference in nodes of the Asc and SN between the right S1‐left thalamus, right S2‐left thalamus, right S1‐right posterior insula, left S1‐right S2, left S1‐left thalamus, right S2‐left S2, left S2‐right thalamus, and right anterior insula‐right dlPFC. Cross‐network static and dynamic theta, beta, and low gamma FCp between SN‐Asc was also negatively correlated with pain interference across right dlPFC and: the right S2, right posterior insula, left posterior insula and the right thalamus as well as the right anterior insula‐left S2. Cross‐network static theta AEC between the DMN‐SN was negatively correlated with pain interference across mPFC‐right anterior insula however the same cross‐network static and dynamic alpha, beta FCp was negatively correlated with pain interference between mPFC‐MCC. Last, cross‐network alpha dynamic wPLI was positively correlated with pain interference between SN‐Desc nodes right anterior insula‐sgACC while dynamic beta wPLI between Asc‐Desc nodes right S1‐sgACC was negatively correlated with pain interference.

In women, the most prominent relationship between FCp and pain interference was observed between cross‐network FCp between SN‐Asc. In this cross‐network FCp, dynamic alpha AEC was positively correlated with pain interference across right TPJ‐right S1 however, the same cross‐network static and dynamic FCp in alpha beta, theta, and low gamma was negatively correlated with pain interference between the right TPJ and: left S1, right S2, right thalamus, and the left thalamus as well as between the right anterior insula and: the right posterior insula, the left posterior insula and the right thalamus. Cross‐network static theta AEC between DMN‐SN was negatively correlated with pain interference across the right TPJ‐PCC, as well DMN‐Asc between the mPFC‐left S2 and between Asc‐Desc across right S1‐sgACC and right posterior insula‐sgACC. Last, pain interference was positively correlated with dynamic beta AEC between the SN‐Desc across right anterior insula‐sgACC.

**FIGURE 8:**
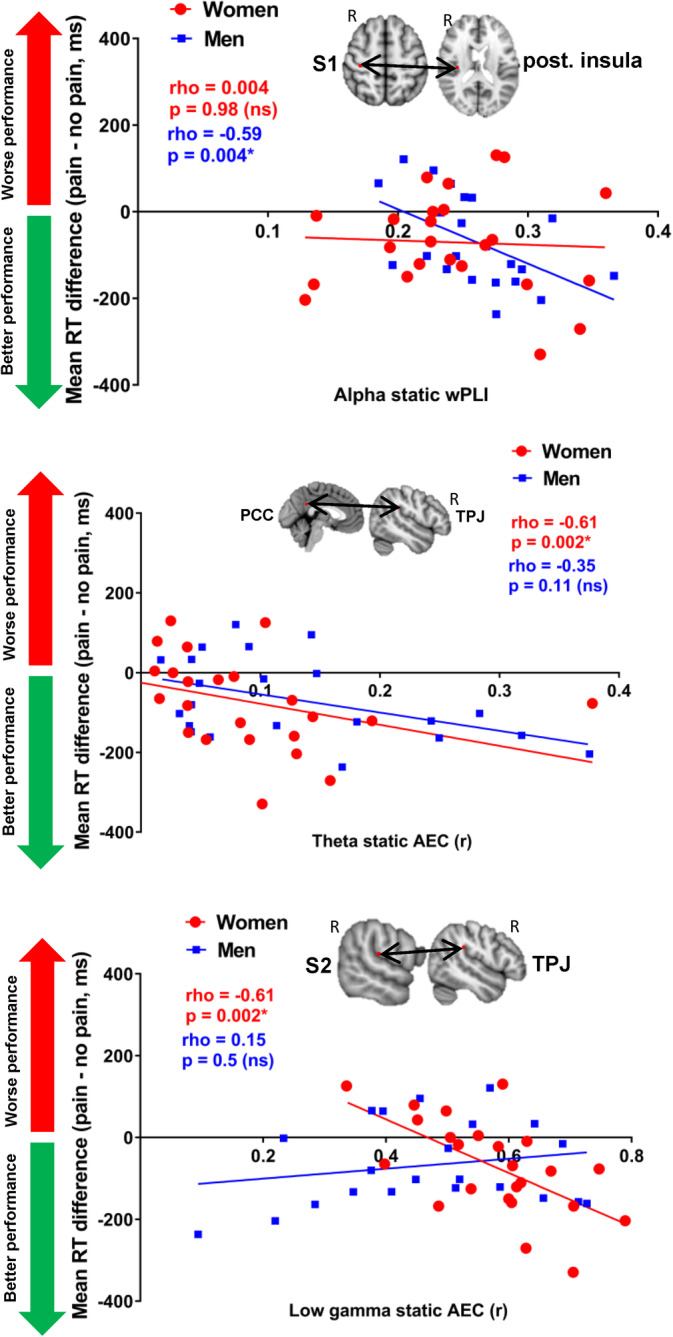
Examples of sex differences in significant correlation between static and dynamic FCp and pain interference. The example nodes which are shown from left to right: within‐network static alpha weighted phase lag index between the right primary somatosensory cortex and the right posterior insula, cross‐network static theta amplitude envelope correlation between the posterior cingulate cortex and the right temporoparietal junction, static low gamma amplitude envelope correlation between the right secondary somatosensory cortex and the right temporoparietal junction. AEC, amplitude envelope correlation; PCC, posterior cingulate cortex; S1, primary somatosensory cortex; S2, secondary somatosensory cortex; TPJ, temporoparietal junction; wPLI, weighted phase lag index

All of the correlation values are summarized on Table 4 and on Figure 4b and examples of correlations are summarized on Figure 8.

**TABLE 4:** Sex differences in correlations between pain interference (A/P score) and FCp

Within‐network	Network nodes	Male A/P (rho)	Female A/P (rho)
Asc	R S1‐L thalamus	−.55 (alpha dwPLI)	Ns
	R S1‐R PI	−.59 (alpha swPLI)	Ns
	L S1‐L thalamus	−.56 (alpha sAEC)	Ns
	L S1‐R S2	−.57 (LG sAEC)	Ns
	R S2‐L S2	−.62 (theta sAEC)	Ns
	R S2‐L PI	−.58 (theta sAEC)	Ns
	R S2‐L thalamus	−.6 (theta swPLI) −.54 (theta dwPLI)	Ns
	L S2‐R thalamus	−.54 (theta dAEC)	Ns
	L S2‐L thalamus	Ns	.53 (alpha dAEC) −.68 (alpha swPLI)
SN	R AI‐R dlPFC	−.55 (alpha swPLI)	Ns

Cross‐network			
Asc‐SN	R S1‐R TPJ	Ns	−.59 (alpha dAEC)
	L S1‐R TPJ	Ns	−.54 (alpha sAEC)
	L S1‐R dlPFC	−.69 (alpha sAEC)	Ns
	R S2‐R dlPFC	−.56 (theta dAEC) −.64 (theta swPLI)	Ns
	R S2‐R TPJ	Ns	−.58 (beta sAEC) −.61 (LG sAEC)
	L S2‐R AI	−.58 (beta sAEC)	Ns
	R PI‐R AI	Ns	−.62 (theta swPLI)
	R PI‐R dlPFC	−.63 (theta sAEC) −.6 (beta sAEC) −.56 (LG sAEC)	Ns
	L PI‐R AI	Ns	−.53 (LG dwPLI)
	L PI‐R dlPFC	−.6 (theta sAEC)	
	R thalamus‐R AI	Ns	−.56 (theta swPLI)
	R thalamus‐R dlPFC	−.58 (theta sAEC)	Ns
	R thalamus‐R TPJ	Ns	−.63 (dwPLI)
	L thalamus‐R TPJ	Ns	−.51 (theta dAEC)
Asc‐DMN	L S2‐mPFC	Ns	.59 (alpha dwPLI)
Asc‐Desc	R S1‐sgACC	−.58 (beta dwPLI)	−.52 (LG dwPLI)
	R PI‐sgACC	Ns	−.54 (theta sAEC)
SN‐DMN	R TPJ‐PCC	Ns	−.61 (theta sAEC)
	R AI‐mPFC	−.59 (theta sAEC)	Ns
	MCC‐mPFC	.62 (alpha dAEC) −.57 (beta swPLI)	Ns
SN‐Desc	R AI‐sgACC	.56 (alpha dwPLI)	.61 (beta dAEC)

*Note:* Significant Pearson's correlations at FDR < .05 are shown for each group.

Abbreviations: AEC, amplitude envelope correlation; AI, anterior insula; Asc, ascending nociceptive pathway; d, dynamic; Desc, descending antinociceptive pathway; dlPFC, dorsolateral prefrontal cortex; DMN, default mode network; L, left; LG, low gamma; MCC, medial cingulate cortex; PI, posterior insula; R, right; s, static; S1, primary sensory cortex; S2, secondary sensory cortex; sgACC, subgenual anterior cingulate cortex; SN, salience network; TPJ, temporoparietal junction.

## 4 DISCUSSION

The current study identified relationships between inherent brain network dynamics and pain sensitivity. To do this, we developed a novel metric to evaluate dynamic functional coupling that provides a window into dynamics of oscillatory neural activity and its relationship with pain. It should be noted that the term “functional coupling” (FCp) refers to a concept related to that of “functional connectivity”, the latter term being used primarily in fMRI studies to designate synchronous activity between brain areas. Here, we report three main findings: First, there is a robust relationship between FCp and pain sensitivity/pain interference. These relationships (summarized in Table 2) were observed across nodes of the Asc and the SN for both within‐ and cross‐ network connections. Second, sex differences in FCp were identified within the dynamic pain connectome; a general pattern being that women exhibited greater static FCp (particularly across nodes of the Asc in the theta and alpha bands) and men exhibiting greater dynamic FCp (particularly across nodes of the SN mainly in the beta and low gamma bands). Last, sex differences in the relationship between FCp and pain sensitivity was most pronounced in men across Asc and SN while sex differences in the relationship between FCp and pain interference were most pronounced in the theta band.

Our study builds on previous findings that identified pain‐evoked oscillatory activity in healthy individuals within a specific functional band (Furman et al., 2017; Huishi Zhang, Sohrabpour, Lu, & He, 2016; May et al., 2019; Nickel et al., 2019; Nir, Sinai, Moont, Harari, & Yarnitsky, 2012; Ploner et al., 2006; Schulz et al., 2015). These previous studies mostly relied on electroencephalography (EEG) and so the findings had limited spatial resolution. As such, a main advantage and novelty of our current study is the use of MEG to specifically interrogate regions of the dynamic pain connectome associated with pain and the focus on inter‐regional oscillatory communication. Furthermore, our use of a resting state paradigm (rather than experimental evoked pain) allowed us to measure trait brain dynamics associated with pain processing. This provides insight into the inherent or “priors” of brain activity that are critically important to set the conditions from which an individual's pain sensitivity likely originated from. Finally, another novel feature of our study was the development and application of a measure of the dynamics of oscillatory communication within the dynamic pain connectome.

At the whole group level, there was a robust association between Asc and SN with pain measures (pain sensitivity, pain interference). Our previous fMRI studies have linked activity of the SN and the Asc with cognitive performance during painful stimulation in healthy controls (Cheng et al., 2017; Erpelding & Davis, 2013; Seminowicz & Davis, 2006, 2007). One of these studies showed that increased dFC within the SN is associated with less pain interference (Cheng et al., 2017). In the current study, we found negative correlations between pain interference and within SN static theta FCp. Negative correlations between pain interference and dynamic FCp were more prominent with dynamic FCp between Asc and SN and within the Asc. Similar patterns of negative correlations were found between Asc‐SN FCp and HPT. The Asc consists of brain regions thought to relay nociceptive input while the salience system is associated with attention toward an incoming stimulus (Craig, 2003; Menon & Uddin, 2010). Interestingly both HPT and pain interference were negatively correlated with the Asc‐SN cross‐network dynamics. Since HPT and pain interference were not correlated with each other, this rules out the possibility that the negative correlation resulted from a link between the two variables. Similar patterns of relationships between Asc‐SN FCp and pain measures may arise because individuals who have greater dynamic engagement between the Asc and SN are able to orient their attention more nimbly to incoming stimuli and the flexibility of their attentional system may allow them to redirect their attention readily. Increased flexibility in the SN may reflect the possibility that in individuals who improve their performance with painful stimulation, painful stimuli improves their performance by increasing their levels of arousal as theorized by the inverted‐U hypothesis of performance and arousal (Landers, 1980). There was a key difference between the brain/pain relationships across the two pain measures: HPT but not pain interference was correlated with cross‐network dynamic FCp between the Asc and the DMN. The DMN is believed to receive sensory information (Raichle, 2015) and is also involved in mind wandering and internal thought (Andrews‐Hanna, 2012; Andrews‐Hanna, Smallwood, & Spreng, 2014; Axelrod, Rees, & Bar, 2017; Kucyi et al., 2013; Kucyi & Davis, 2014). As such, increased dynamics between the DMN and the Asc may result in less mind wandering away from a stimulus and increased sensitivity toward incoming stimuli resulting. Thus, a reduced HPT could be the outcome of individual differences in cross‐network FCp.

We previously used fMRI to demonstrate that there are sex differences of the connectivity of the sgACC with the SN and the Desc networks (Wang et al., 2014). The current study builds on this finding of sex differences within the dynamic pain connectome using the millisecond temporal resolution of MEG. Our most pronounced sex difference here was that women had greater static FCp mainly in the theta and alpha bands while men had greater dynamic FCp mainly in the beta and low gamma bands. Sex differences in static FCp was observed across nodes of the SN and across nodes of the Asc. Sex differences involving the SN was expected based on our previous fMRI findings (Wang et al., 2014) but we did not expect to find sex differences of the Asc, although sex differences have been reported in the functional connectivity of the left and the right somatosensory cortices (H. Liu, Stufflebeam, Sepulcre, Hedden, & Buckner, 2009). Our results showed that inter‐hemispheric static FCp in the Asc and between Asc‐SN were significantly different between the sexes. Other sex‐differences findings observed with dFCp were particularly interesting because many of the findings involved the sgACC. The sgACC and the Desc have demonstrated sex‐differences of functional connectivity in several past studies (Biswal et al., 2010; Kong, Tu, Zyloney, & Su, 2010; Wang et al., 2014). Thus, our current findings support and extend previous findings (Casanova, Whitlow, Wagner, Espeland, & Maldjian, 2012) and suggest that dynamic cross‐network FCp involving the sgACC show widespread sex differences. Thus even at high temporal resolutions (beta, low gamma dynamic FCp) the sgACC can exhibit activity that indicates is an important hub for sex differences in brain mechanisms of pain processing.

Utilizing the high temporal resolution of the MEG, we had the opportunity to examine whether there are sex‐specific patterns of FCp. We found that the majority of sex differences in static FCp were observed in the theta and alpha bands, whereas sex differences in dynamic FCp were observed in the beta and low gamma bands. Frequency bands are thought to serve specific functions in network level organization (Buzsaki, 2006; Buzsáki & Draguhn, 2004). In particular, slow frequency bands (e.g., alpha and theta) are thought to be involved in long range communication and network level organization, whereas faster frequency bands (e.g., gamma) have been associated with local organization of functions. As such, the sex differences we found for static FCp in the slower frequency bands may be attributable to inherent differences in network organization between men and women. In contrast, our findings of sex differences in dynamic FCp may reflect more transient differences in the local organization of brain regions within the dynamic pain connectome because it has previously been shown that more diverse states are occupied in men (Yaesoubi, Miller, & Calhoun, 2015).

The sex differences we identified in the relationship between pain and the brain demonstrate that there are both common and unique sets of networks related to pain sensitivity and pain interference in men and women. The most robust relationship in both sexes was observed between the pain measures and cross‐network FCp across Asc‐SN. However, sex differences were most prominent in the relationships with within‐network FCp in the Asc and the SN where significant relationships with pain measures were mostly observed in men. In women, many of the significant relationships were observed with cross‐network FCp, especially in the theta band. These results suggest pain processing may depend more on the dynamics of within‐network connections in men and on cross‐network connections in women. In addition, sex differences in the spectral profile of the significant relationships between pain measures and FCp suggest that there may be different underlying mechanisms (e.g., local organization vs. global organization of networks) related to pain processing in men and women.

In conclusion, the current study sheds light on brain mechanisms of pain processing in healthy men and women. Individual variability in pain processing may be explained dynamic interaction between regions of the dynamic pain connectome through neural oscillations however, the regions involved in theses interactions vary widely across the canonical bands. Thus, the temporal aspects of dynamic neural interactions are crucial to healthy pain processing. Furthermore, men and women had distinct functional band involvement in the dynamic neural interactions associated with pain processing suggesting sex differences in the underlying brain mechanisms of pain processing. Thus the type of frequency band and the different brain networks involved in the dynamic integration of brain regions play an important role in pain processing of men and women.

## CONFLICT OF INTEREST

The authors have no conflict to declare.

## Data Availability

Data pertaining to the results of this study may be obtained through the corresponding author upon reasonable request.
